# Bacterial defense and phage counterdefense lead to coexistence in a modeled ecosystem

**DOI:** 10.1073/pnas.2414229121

**Published:** 2024-10-25

**Authors:** Ofer Kimchi, Yigal Meir, Ned S. Wingreen

**Affiliations:** ^a^Lewis-Sigler Institute for Integrative Genomics, Princeton University, Princeton, NJ 08544; ^b^Department of Physics, Ben-Gurion University, Be’er Sheva 84105, Israel; ^c^Department of Physics, Princeton University, Princeton, NJ 08544; ^d^Department of Molecular Biology, Princeton University, Princeton, NJ 08544

**Keywords:** bacteria, phage, ecology, model, coexistence

## Abstract

Bacteria have evolved many defenses against invading viruses (phage). Despite the many bacterial defenses and phage counterdefenses, in most environments, bacteria and phage coexist, with neither driving the other to extinction. How is coexistence realized in the context of the bacteria/phage arms race, and how are immune repertoire sizes determined in conditions of coexistence? Here we develop a simple mathematical model to consider the evolutionary and ecological dynamics of competing bacteria and phage with different immune/counterimmune repertoires. We find an ecologically stable fixed point exhibiting coexistence, in agreement with the experimental observation that each individual bacterium typically carries multiple defense systems, though fewer than the maximum number possible. However, in simulations, the populations typically remain dynamic, exhibiting chaotic fluctuations around this fixed point. These dynamics enable coexistence even when phage (predator) strains outnumber bacteria (prey) strains. We obtain quantitative predictions for the mean, amplitude, and timescale of these dynamics. Our results provide a framework for understanding the evolutionary and ecological dynamics of the bacteria/phage arms race and demonstrate how bacteria/phage coexistence can stably arise from the coevolution of bacterial defense systems and phage counterdefense systems.

Bacteria and the viruses that infect them (phage) have been engaged in an arms race spanning eons. Each bacterium typically carries many defense systems to protect against phage (1). Simultaneously, phage counterdefense systems enable them to evade these bacterial defenses (2). Both bacterial defense systems and phage counterdefense systems impose a fitness cost on the strains that carry them (3, 4). In bacteria, for example, both metabolic costs associated with protein production (5) as well as inadvertent self-targeting (6) contribute to the fitness cost. In the absence of selection pressure from the opposing species, these systems are therefore quickly lost, over the timescale of a few generations (7). We sought to understand the coevolution of these systems. What determines the size of a bacterial cell’s immune repertoire and a single phage’s counterimmunity repertoire?

The multiplicity of defense systems may enable bacteria to repel phage invaders by spreading defense systems through horizontal gene transfer (8). This ‘pan-immunity hypothesis’ explains boom and bust cycles of phage and bacteria as arising from subsequent rounds of phage invasion and bacterial defense. In contrast, we wanted to study how persistent coexistence of phage and bacteria is realized within the context of the defense/counterdefense arms race.

We consider a mixture of bacterial strains with population densities *B*_*i*_ and phage strains with densities *P*_*j*_. Each bacterial strain carries a subset of a total number $n^{\text{tot}}$ possible defense systems. Bacterial strains carrying more systems have a smaller growth rate *α*_*i*_. Similarly, each phage strain carries a set of counterdefense systems, such that phage *j* can infect bacteria *i* if it has a corresponding counterdefense system for each of bacteria *i*’s defense systems (9). The cost of counterdefense systems is imposed on the phage burst size $b_{j}+1$. Bacterial and phage strain death rates are given by *μ*_*i*_ and *δ*_*j*_, respectively. Finally, we include steady immigration fluxes *λ*_*i*_ and *ν*_*j*_ for the bacteria and phage. The system’s dynamics are given by

[1] $$\begin{array}{c} \frac{dB_{i}}{\mathrm{dt}}=B_{i}(\alpha_{i}-\mu_{i}-\sum_{j}k_{\mathrm{ij}}P_{j})+\lambda_{i};\;\;\;\frac{dP_{j}}{\mathrm{dt}}=P_{j}(-\delta_{j}+b_{j}\sum_{i}k_{\mathrm{ij}}B_{i})+\nu_{j}, \end{array}$$

where the infection rate $k_{\mathrm{ij}}=k$ if phage *j* can infect bacteria *i* and 0 otherwise, and where we have neglected delays associated with phage reproduction (10). For simplicity, we focus on the case where each defense/counterdefense system has the same cost, and the immigration and death rates are strain-independent. We define a “largest positive-growth repertoire size” for bacteria and phage, such that bacteria with $n_{b}>n_{b}^{\text{pos}}$ defense systems have a negative net growth rate *α* − *μ*, and phage with $n_{p}>n_{p}^{\text{pos}}$ counterdefense systems have a negative net burst size *b*.

We first address Eqs. **1** analytically. For a system composed exclusively of bacteria with *n*_*b*_ defense systems and phage with *n*_*p*_ counterdefense systems, a dynamically stable fixed point (dfp) can be found by solving $\frac{dB_{i}}{\mathrm{dt}}=\frac{dP_{j}}{\mathrm{dt}}=0$. Due to symmetry, at this dfp, all bacterial strains with *n*_*b*_ defense systems are present at equal population densities $B_{n_{b}}^{\text{fp}}$, and all phage strains with *n*_*p*_ counterdefense systems are present at equal population densities $P_{n_{p}}^{\text{fp}}$. For certain combinations $\left(n_{b}^{⋆},n_{p}^{⋆}\right)$, this dfp will also be an ecologically stable fixed point (efp) with respect to invasions by bacteria with $n_{b}^{⋆}\pm 1$ defense systems, and to phage with $n_{p}^{⋆}\pm 1$ counterdefense systems (*SI Appendix*, sections S1 and S2). At this efp, bacteria typically carry fewer than the maximal number of defense systems allowed by a positive growth rate, and similarly phages carry fewer than the maximal number of counterdefense systems (Fig. 1*A*).

**Fig. 1.**
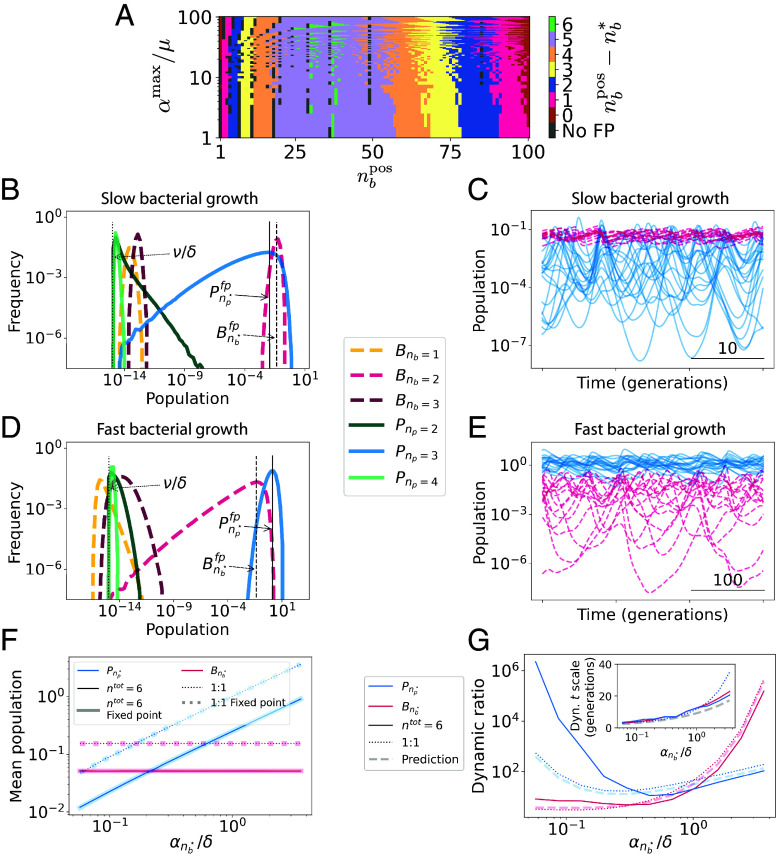
Fixed-point and dynamical simulation results. (*A*) Predicted ecological fixed point for $n^{\text{tot}}=110$ total defense systems, with $n_{p}^{\text{pos}}=100$ maximum counterdefense systems per phage strain. Distance between predicted ecological fixed point $n_{b}^{⋆}$ and maximal number of defense systems per bacterial strain, $n_{b}^{\text{pos}}$, is shown as a function of $n_{b}^{\text{pos}}$ and the ratio between the growth rate of bacteria with no defense systems, $\alpha^{\text{max}}$, and the death rate of bacteria, *μ*. Gray points represent parameters for which no ecologically stable fixed point is predicted. (*B*) Representative simulation results for an $n^{\text{tot}}=6$ system with $\alpha^{\text{max}}/\delta =0.1$ and $\alpha_{n_{b}^{⋆}}/\delta \approx 5.7\times 10^{-2}$, displaying the histogram of population densities of bacterial and phage strains with different numbers of defense/counterdefense systems per strain. (*C*) Population dynamics of bacteria with $n_{b}=n_{b}^{⋆}=2$ defense systems each (red) and phage with $n_{p}=n_{p}^{⋆}=3$ counterdefense systems each (blue) in the $n^{\text{tot}}=6$ simulation of panel (*B*). (*D* and *E*) Representative simulation results as in (*B* and *C*), but with bacterial growth rate increased to $\alpha^{\text{max}}/\delta =10$ (and $\alpha_{n_{b}^{⋆}}/\delta \approx 5.7$). (*F*) Dependence of mean population densities for $n^{\text{tot}}=6$ system (narrow solid curves), $n^{\text{tot}}=0$ system (narrow dotted curves), and analytical dynamical fixed-point prediction from **Eq. 1** (corresponding wide curves). (*G*) Dependence of dynamic ratio (main panel) and timescale of dynamics (*Inset*) for $n^{\text{tot}}=6$ system (solid curves), $n^{\text{tot}}=0$ system (dotted curves), and analytical prediction (*SI Appendix*, Eq. **S10**; dashed curves). Here, initial conditions were kept constant independent of *x*-axis parameter variation. The dynamic ratio measures the amplitude of fluctuations and is defined as the typical ratio of local maxima to local minima. See *SI Appendix*, sections S3 and S4 for further discussion.

We next turn to dynamical simulations. We consider a system with $n^{\text{tot}}=6$ possible defense/counterdefense systems, with parameters such that $n_{b}^{\text{pos}}=3$ and $n_{p}^{\text{pos}}=4$, and for which we predict the existence of an efp with $\left(n_{b}^{⋆},n_{p}^{⋆}\right)=\left(2,3\right)<\left(n_{b}^{\text{pos}},n_{p}^{\text{pos}}\right)$ (*SI Appendix*, Eq. **S5**). At this efp, both bacterial growth rate and phage burst size are roughly half their maximum values. After a short transient period, we find that all populous bacterial strains have $n_{b}^{⋆}$ defense systems, and all populous phage have $n_{p}^{⋆}$ counterdefense systems (Fig. 1*B*). In the absence of immigration (λ=ν=0) only these strains survive. The analytically predicted dfp population densities predict well the time-averaged population densities of these strains (Fig. 1*B*, vertical lines). However, the system displays persistent chaotic dynamics (Fig. 1*C*; Lyapunov exponent = 0.08; see *SI Appendix*, section S3) (11).

These chaotic population fluctuations enable coexistence to be maintained even when the number of phage “predator” strains, $\left(\begin{array}{c} n^{\text{tot}}\\ n_{p}^{⋆} \end{array}\right)$, exceeds the number of bacterial “prey” strains $\left(\begin{array}{c} n^{\text{tot}}\\ n_{b}^{⋆} \end{array}\right)$ (12, 13). For different parameter choices, the chaotic fluctuations enabling this coexistence may either be largest in the phage population (as in Fig. 1 *B* and *C*) or largest in the bacterial population (as in Fig. 1 *D* and *E*). This variation in the dynamics comes with no apparent effect on the coexistence itself: we find that all bacteria with $n_{b}^{⋆}$ defense systems and all phage with $n_{p}^{⋆}$ counterdefense systems coexist in all cases we examined, even in the absence of immigration, λ=ν=0. Sufficient parameter heterogeneity can lead some strains to go extinct, but we found no extinction within $10^{6}$ generations for moderate amounts of parameter heterogeneity $(O\left(10^{-5}\right)$).

While the average population densities are well predicted by the dfp population densities (Fig. 1*F*), the dynamics cannot be predicted by fixed-point analysis. Helpfully, we find a quantitatively similar parameter dependence of dynamics in the analytically tractable $n^{\text{tot}}=0$ case, corresponding to a minimal oscillatory system of one bacterial strain and one phage strain (“1:1 case”; see *SI Appendix*, section S4). Both the parameter-dependent dynamic ratio (i.e., the amplitude of fluctuations) and the dynamics’ timescale are in quantitative agreement between the $n^{\text{tot}}=6$ and 1:1 cases (Fig. 1*G*). Using the 1:1 system as a guide, we can thus predict the parameter-dependent features of the chaotic dynamics for more complex cases with multiple bacterial and phage strains.

Our model makes several simplifying assumptions. While we have considered all defense systems to be qualitatively interchangeable, defense systems in nature operate through different mechanisms and may have qualitatively different effects on both the growth cost to the bacterium and on the success or failure of the invading phage (and similarly for phage counterdefense systems) (3). Interactions among defense systems may also change their efficacies (14, 15). We have also focused exclusively on obligate lytic (virulent) phage, neglecting alternative phage infection strategies (16). Although temperate phage may qualitatively affect the behavior of many phage–bacteria interactions (17), here, the effect of superimmunity exclusion (i.e., that lysogens are immune to infection by phage of the same strain that lysogenized them) may be considered as a special case of a defense system. Similarly, chronic infections wherein phages reproduce and exit the cell without cell lysis may be considered as a modification of the infection rate *k* and the burst size *b*. Furthermore, although we have assumed well-mixed populations, spatial organization likely affects both the dynamics and coexistence of phage/bacteria communities, especially in nonaquatic environments (18). In this regard, abortive infection defenses will be a fruitful topic for future work. Finally, a variant of the model described by Eqs. **1** without immigration but allowing strains to stochastically gain, lose, and exchange systems with one another through mutations and horizontal gene transfer, yields qualitatively similar results (*SI Appendix*, section S3).

In summary, we have developed a model for the dynamics of competing phage and bacteria with different sets of defense and counterdefense systems. A fixed-point analysis (confirmed by dynamical simulations) indicates that phage and bacteria typically evolve to have more than one and fewer than the maximum number of defense/counterdefense systems in each strain. This qualitative behavior has been observed in nature and has previously been explained by the pan-immunity hypothesis, which argues that invading phage can be driven to extinction as long as some bacteria within a community are immune to the invading phage, and that this immunity can be conferred to other bacteria through horizontal gene transfer (8). In contrast, we find that within our model, large immunity and counterimmunity repertoires emerge naturally and enable bacteria/phage coexistence. This coexistence is manifested in persistent chaotic dynamics, with the mean, amplitude, and timescale of these dynamics well predicted by an analysis based on the competition between a single bacterial and a single phage strain. Although the ecological fixed point of the system $\left(n_{b}^{⋆},n_{p}^{⋆}\right)$ depends on the details of defense and counterdefense system costs (*SI Appendix*, Eq. S5), we find that the qualitative dynamics depend only on the ratio α/δ (*SI Appendix*, Eqs. **S13** and **S18**). Thus, there are two main regimes of system behavior: compared to phage death rate, the bacterial growth rate may either be slow (Fig. 1 *B* and *C*) or fast (Fig. 1 *D* and *E*). Finally, although we have focused our analytical analysis on symmetric systems, our simulations demonstrate that even nonsymmetric systems with modest amounts of heterogeneity are not limited by the principle of competitive exclusion in our model. Thus, we find that the chaotic dynamics of the system enable the coexistence of more phage (predator) strains than bacterial (prey) strains, exceeding the biodiversity predicted by other frameworks such as “kill-the-winner” (19).

The discovery that bacteria typically carry multiple coexisting defense systems (and phage, multiple counterdefense systems) has raised many questions. Chief among these, what controls the number and type of defense and counterdefense systems in a particular bacterium or phage? One possibility is that these systems are controlled by happenstance, with horizontal gene transfer mediating random gains and losses of systems. Alternatively, our simple model suggests that states of evolutionary stability—albeit with fluctuations about these states—may provide a helpful guiding perspective.

## Materials and Methods

Simulations were performed using Python version 3.9.13. Details of derivations and simulation methodology may be found in *SI Appendix*.

## Supplementary Material

Appendix 01 (PDF)

## Data Availability

There are no data underlying this work.
