# Supplementary material for: Bacterial defense and phage counterdefense lead to coexistence in a modeled ecosystem
Source: Proc Natl Acad Sci U S A. 2024 Oct 25;121(44):e2414229121. doi: 10.1073/pnas.2414229121 (PMC11536147; doi:10.1073/pnas.2414229121)
Supplement: Supplementary file 1 — Appendix 01 (PDF) [file pnas.2414229121.sapp.pdf]

Supplement: Bacterial defense and phage counter-defense lead to coexistence in a modeled ecosystem

S1 Deriving the conditions for an ecologically stable fixed point

To find the conditions defining an ecologically stable fixed point, we start from Eqs. 1, making the simplifications discussed in the main text, and assuming that the immigration terms λ and ν are negligible for the purpose of the following analyses. We consider a system composed exclusively of bacteria with n_b defense systems and phage with n_p counter-defense systems. At a dynamical fixed point of such a system, due to symmetry, all $\binom{n^{\text{tot}}}{n_b}$ bacterial strains with n_b defense systems are present at equal population densities $B^{\text{fp}}_{n_b}$, and all $\binom{n^{\text{tot}}}{n_p}$ phage strains with n_p counter-defense systems are present at equal population densities $P^{\text{fp}}_{n_p}$. This dynamical fixed point is given by

$$\begin{aligned} B^{\text{fp}}_{n_b} &= \frac{\delta}{kb_{n_p}\binom{n_p}{n_b}}, \\ P^{\text{fp}}_{n_p} &= \frac{\alpha_{n_b}-\mu}{k\binom{n^{\text{tot}}-n_b}{n_p-n_b}}, \end{aligned} \tag{S1}$$

where $\binom{n_p}{n_b}$ is the number of bacterial strains each phage strain can infect, and $\binom{n^{\text{tot}}-n_b}{n_p-n_b}$ is the number of phage strains that can infect each bacterial strain. α_{n_b} is the growth rate of bacteria with n_b defense systems, and is given by $\alpha_{n_b} = g(n_b)$ with a monotonically decreasing growth function g. Similarly, $b_{n_p}+1$ is the burst size of phage carrying n_p counter-defense systems, and is given by $b_{n_p}+1 = h(n_p)$ for a monotonically-decreasing function h. (Note that the effective burst size b takes into account the loss of the infecting phage particle). We assume g and h are concave functions, but our results largely hold if they are convex (except in the $n^{\text{tot}} \to \infty$ limit; see Supplementary Section S2).

This dynamical fixed point will further be an ecological fixed point if it is stable to invasions by bacteria with a different number of defense systems, or to phage with a different number of counter-defense systems. This will occur if the growth rates of such invading strains at the dynamical fixed point are negative. We refer to such an ecologically stable fixed point as $(n_b^\star, n_p^\star)$. The growth rate of an invading bacterial strain with $n_b^\dagger$ defense systems at the ecological fixed point would be

$$\frac{dB_{n_b^\dagger}}{dt} = B_{n_b^\dagger}\left(\alpha_{n_b^\dagger} - \mu - k\binom{n^{\text{tot}}-n_b^\dagger}{n_p^\star - n_b^\dagger}P^{\text{fp}}_{n_p^\star}\right), \tag{S2}$$

where $B_{n_b^\dagger}$ is the population of the invading bacterial strain. Similarly, the growth rate of an invading phage strain with $n_p^\dagger$ counter-defense systems would be

$$\frac{dP_{n_p^\dagger}}{dt} = P_{n_p^\dagger}\left(-\delta + kb_{n_p^\dagger}\binom{n_p^\dagger}{n_b^\star}B^{\text{fp}}_{n_b^\star}\right). \tag{S3}$$

These terms are negative for invading bacterial strains with $n_b^\dagger = n_b^\star \pm 1$ defense systems, and for invading phage strains with $n_p^\dagger = n_p^\star \pm 1$ counter-defense systems, when

$$
\begin{aligned}
\frac{\alpha_{n_b^\star+1}-\mu}{\alpha_{n_b^\star}-\mu} &< \frac{n_p^\star - n_b^\star}{n^{\text{tot}}-n_b^\star},\\
\frac{\alpha_{n_b^\star}-\mu}{\alpha_{n_b^\star-1}-\mu} &> \frac{n_p^\star - n_b^\star+1}{n^{\text{tot}}-n_b^\star+1},\\
\frac{b_{n_p^\star+1}}{b_{n_p^\star}} &< \frac{n_p^\star - n_b^\star+1}{n_p^\star+1},\\
\frac{b_{n_p^\star}}{b_{n_p^\star-1}} &> \frac{n_p^\star - n_b^\star}{n_p^\star}.
\end{aligned}
\tag{S4}
$$

By taking the natural logarithm and substituting derivatives for differences, we arrive at the simplified expression

$$
\begin{aligned}
n_p^\star &= n^{\text{tot}}\left[1+\frac{d}{dn_b}\log\left(\frac{\alpha_{n_b}}{\mu}-1\right)\right]\Bigg|_{n_b=n_b^\star},\\
n_b^\star &= -n_p^\star\left[\frac{d}{dn_p}\log\left(b_{n_p}\right)\right]\Bigg|_{n_p=n_p^\star}.
\end{aligned}
\tag{S5}
$$

S2 Ecological fixed point behavior as $n^{\text{tot}} \to \infty$

The behavior of the ecological fixed point defined by Eqs. (S5) as $n^{\text{tot}} \to \infty$ depends on how n_b^{pos} and n_p^{pos} scale with n^{tot}. Since ecological stability requires $n_p^\star > n_b^\star$, $n_p^{\text{pos}} > n_b^{\text{pos}}$ must hold to avoid the trivial outcome with no phage present. This inequality is biologically reasonable given the complexity required of defense systems compared to the relative simplicity of counter-defense systems; as an example, consider the immensely complex Type 1-F CRISPR-Cas system which can be evaded by phage that express a single short RNA molecule [S1]. As $n^{\text{tot}} \to \infty$, there are therefore three possibilities: 1) n_b^{pos} and n_p^{pos} are both intensive; 2) n_b^{pos} and n_p^{pos} are both extensive; 3) n_b^{pos} is intensive while n_p^{pos} is extensive. We find that in cases (1) and (2), $n_b^\star$ and $n_p^\star$ are approximately equal to n_b^{pos} and n_p^{pos}, respectively, as $n^{\text{tot}} \to \infty$. However, surprisingly, in case (3) and for concave cost functions g and h, $n_b^{\text{pos}} - n_b^\star$ and $n_p^{\text{pos}} - n_p^\star$ both grow with n^{tot}. In this case, each individual bacterium optimally carries a subset of possible defense systems, even though it could carry far more and still continue to grow (and similarly for phage).

S3 Details of dynamical simulations

We use the following parameters in our simulations, with time units such that $\delta = 1$ and population density units such that $k = 1$: $\mu = 10^{-2}$; $\lambda = \nu = 10^{-15}$; $b^{\text{max}} = 15$. Bacterial growth rate α is varied as described in the main text; all other parameters are kept constant throughout. The bacterial death rate $\mu = 10^{-2}$ was chosen to be much smaller than the phage death rate δ so that we can explore the $\alpha < \delta$ regime while maintaining $\mu \ll \alpha$, such that bacterial populations are primarily limited by phage predation. For example, for $\alpha_{n_b^*} \approx 0.05$ as in Fig. 1b,c, the rate of bacterial death due to phage predation at the dynamical fixed point, $\binom{n^{\text{tot}}-n_b^\star}{n_p^\star-n_b^\star} k P^{\text{fp}}_{n_p^\star}$, is approximately $5\times$ larger than μ; for $\alpha_{n_b^*} \approx 5$ as in Fig. 1d,e, it is approximately $500\times$ larger. The particular values of λ and ν matter very little as long as they are in the regime of slow immigration. (The opposite regime where immigration is substantial can be qualitatively different because immigration can stabilize the populations of phage and bacteria strains which would otherwise go extinct). Finally, the particular value of the phage burst size b has very little effect on our results since the ecological fixed point depends on $\frac{d}{dn_p}\log b_{n_p}$ (and is therefore unchanged when b is modified by a multiplicative factor; Eq. (S5)) and the qualitative dynamical behavior is mostly determined by α/δ as described in Section S4. Natural phage burst sizes are typically of $\mathcal{O}(100)$ phage particles per burst, but also are accompanied by a sizeable time delay between phage infection and lysis. Given that our model neglects this time delay, the effective burst size must be decreased to reproduce overall phage proliferation rates. $b^{\text{max}} = 15$ was therefore chosen to correspond to a burst size of ~ 200 for a system with a typical lysis time [S2].

We implement concave cost functions $g(n_b) = \alpha^{\max} \cos\left(\frac{\pi n_b}{2n_b^{\max}}\right)$ and $h(n_p) = b^{\max} \cos\left(\frac{\pi n_p}{2n_p^{\max}}\right)$. $n_b^{\max}$ sets the maximum number of defense systems bacteria can have before their growth rate α reaches zero, and is somewhat larger than n_b^{pos} which is determined by the net growth rate $\alpha - \mu$ reaching zero (and similarly for $n_p^{\max}$ for phage). Strains with more systems than the maximum don't grow: $g(n_b \geq n_b^{\max}) = 0$, and similarly, $h(n_p \geq n_p^{\max}) = 0$. In our simulations, we set $n_b^{\max} = 3.27$, and $n_p^{\max} = 4.5$, as these values yielded $(n_b^\star, n_p^\star) = (2, 3)$ and $(n_b^{\text{pos}}, n_p^{\text{pos}}) = (3, 4)$ for our parameter choices.

In all simulations, the growth rates of individual bacterial strains α_i, the burst sizes of individual phage strains b_j, and the infection rates k_{ij} were all varied at order 10^{-5}. Specifically, these terms were multiplied by $\left(1 + 10^{-5} \times (2r - 1)\right)$ where r is a random number uniformly distributed between 0 and 1, chosen randomly and independently for each strain or strain-strain interaction. Thus, small amounts of parameter heterogeneity are added to the system; for example, values of k ultimately range from $(1 - 10^{-5})$ to $(1 + 10^{-5})$.

We ran each simulation for 10^7 time units (equivalent to 1.4×10^6 bacterial generations for $\alpha/\delta = 0.1$, or to more generations for larger α).

We also ran simulations initializing the system with only bacterial strains for which $n_b = n_b^\star$ and phage strains with $n_p = n_p^\star$, and setting the immigration fluxes to zero, $\lambda = \nu = 0$. We found qualitatively and quantitatively similar results to those displayed in Fig. 1b-e, and observed no phage or bacterial strains going extinct over the course of these simulations. The simulations in Fig. 1f-g were performed in this manner.

To measure the Lyapunov exponent, we initialized two trajectories using the parameters of Fig. 1b,c, with initial values of phage populations $\sim 10^{-14}$ higher in one trajectory than the other. Specifically, the initial values in the second simulation were equal to those of the first, plus $10^{-14} \times (2r - 1)$, where r is a uniformly distributed random number between 0 and 1 chosen independently for each phage strain. We then measured the distance between the two trajectories as $\sqrt{(B - B')^2}$ where B is the total bacterial population. This value grows exponentially until ~ 400 generations, at which point it begins fluctuating between 10^{-5} and 10^0. This behavior of the distance between trajectories initialized nearby (namely, exponential growth, followed by fluctuations around a fixed value) is typical of chaotic systems. The Lyapunov exponent is defined as the slope of the exponential growth segment. To measure this, the logarithm of the distance was fit to a line (or equivalently, the distance was fit to an exponential). This analysis was repeated ten times for different instantiations of r, with measurements of the Lyapunov exponent having a mean of 0.0822 and a standard deviation of 0.0014.

In Fig. 1g, we needed to measure the dynamic ratio in a consistent way for both chaotic and oscillatory systems. To do this, first, we identified the local peaks and troughs (i.e. where the derivatives of the population densities change sign). For oscillatory systems, the peaks are all at (very nearly) equal values, as are the troughs. For chaotic systems, this is not the case. In order to measure the peaks and troughs in a manner that does not change much between different instantiations of the chaotic dynamics, we therefore defined the dynamic ratio as the ratio of the 90th percentile of the local population density peaks to the 10th percentile of the local population density troughs. The dynamic timescale was measured by taking the median of the time between local maxima and the median of the time between local minima, and averaging these two medians. For oscillatory systems, this measures the period of oscillations.

The simulations in Fig. 1f-g were initiated at $B_i = 0.06 \times (1 + r)$ and $P_j = 0.06 \times (1 + r)$ where r is a random number uniformly distributed between 0 and 1, chosen independently for each bacterial and each phage strain. Qualitatively similar results were obtained for simulations initiated at $B_i = B^{\text{fp}}_{n_b^\star} \times (1 + r)$ and $P_j = P^{\text{fp}}_{n_p^\star} \times (1 + r)$, i.e. where the initial conditions depend on parameter values through the dynamical fixed-point population densities.

Finally, we developed a variant of these simulations to take into account the stochasticity of evolutionary dynamics. Rather than initiating the system with all strains present, we initialized the system with only one bacterial strain (with one defense system), and one phage strain (with the corresponding counter-defense system). After every 5 timepoints of simulation, we performed a mutation step. In this mutation step, new bacterial or phage strains could be created, by either gaining or losing defense or counter-defense systems. First, we choose whether to mutate the bacteria (with probability p) or the phage (with probability $1 - p$); we chose $p = 10^{-2}$ to approximate the challenge bacteria face in constructing new defense systems as opposed to the relative simplicity of phage counter-defense systems, as discussed in the previous section. Next, we select the strain to mutate, proportionally to its population density at the time of the mutation step. We then determine whether the mutation will be the (a) gain or (b) loss of defense (or counter-defense) systems, each occurring with probability $1/2$. Finally, all possible single mutants either adding or removing a defense or counter-defense system (depending on which was selected) from the selected strain are added to the system at a small initial population (10^{-15}), or if they were already present, their population is increased by the same small amount. These simulations resulted in the same qualitative behavior as the constant immigration rate simulations

discussed in the main text, evolving towards the ecologically stable fixed point at the system level, and exhibiting chaotic dynamics at the population level.

S4 Dynamical analysis

To understand the dynamical behavior of the system, we turn to the $n^{\text{tot}} = 0$ case, which we refer to as the 1:1 case since it involves a single bacterial strain and a single phage strain. In Fig. 1f-g, we show that certain aspects of the dynamical behavior of the 1:1 case closely parallel those of systems with $n^{\text{tot}} > 0$. In this section, we quantitatively analyze the 1:1 case.

First, we perform linear stability analysis to describe the behavior of the system near the dynamical fixed point (Eqs. (S1)). The essential element of linear stability analysis is the calculation of the eigenvalues of the Jacobian matrix at the dynamical fixed point. For the 1:1 case, the eigenvalues are $\pm i\sqrt{\delta\,(\alpha-\mu)}$. That these are imaginary indicates that the 1:1 case undergoes continued oscillations. The period of these oscillations is given by $2\pi/\sqrt{\delta\,(\alpha-\mu)}$. This prediction is plotted as a dashed gray curve in the inset to Fig. 1g.

Predicting the amplitude of oscillations is less straightforward, and to our knowledge no generic method enables this prediction. Linear stability analysis provides no information regarding the amplitude of oscillations. To address this challenge, we recognize that there is a quantity C satisfying $dC/dt = 0$. In general, this quantity is given by

$$C = P^{\text{fp}}_{n_p} \sum_j \left(\frac{P_j(t)}{P^{\text{fp}}_{n_p}} - \log \frac{P_j(t)}{P^{\text{fp}}_{n_p}} \right) + bB^{\text{fp}}_{n_b} \sum_i \left(\frac{B_i(t)}{B^{\text{fp}}_{n_b}} - \log \frac{B_i(t)}{B^{\text{fp}}_{n_b}} \right). \tag{S6}$$

For the 1:1 case, this quantity simplifies to

$$C = P^{\text{fp}}_0 \left(\frac{P(t)}{P^{\text{fp}}_0} - \log \frac{P(t)}{P^{\text{fp}}_0} \right) + bB^{\text{fp}}_0 \left(\frac{B(t)}{B^{\text{fp}}_0} - \log \frac{B(t)}{B^{\text{fp}}_0} \right), \tag{S7}$$

where P^{fp}_0 and B^{fp}_0 are given by Eqs. (S1) with $n_p = n_b = n^{\text{tot}} = 0$.

Because C is a constant in time, it will also be a constant when P or B are at an extremum. Solving for B at either extremum of P (i.e. where $dP/dt = 0$) yields $B = B^{\text{fp}}_0$. Similarly, solving for P at either extremum of B yields $P = P^{\text{fp}}_0$. Thus, we find that

$$\begin{aligned} C &= P^{\text{ext}} - P^{\text{fp}}_0 \log \frac{P^{\text{ext}}}{P^{\text{fp}}_0} + bB^{\text{fp}}_0 \\ &= bB^{\text{ext}} - bB^{\text{fp}}_0 \log \frac{B^{\text{ext}}}{B^{\text{fp}}_0} + P^{\text{fp}}_0, \end{aligned} \tag{S8}$$

where B^{ext} represents the value of $B(t)$ at its maximum or minimum, and similarly for P^{ext}.

We then estimate the values of P^{min}, P^{max}, B^{min}, and B^{max}. For the minimum values, we treat the linear terms (e.g. P^{ext}) as negligible compared to the logarithmic terms; for the maximum values, we treat the logarthmic terms as negligible. These approximations yield

$$\begin{aligned} C &= P^{\text{max}} + bB^{\text{fp}}_0, \\ &= -P^{\text{fp}}_0 \log \frac{P^{\text{min}}}{P^{\text{fp}}_0} + bB^{\text{fp}}_0, \\ &= bB^{\text{max}} + P^{\text{fp}}_0, \\ &= -bB^{\text{fp}}_0 \log \frac{B^{\text{min}}}{B^{\text{fp}}_0} + P^{\text{fp}}_0. \end{aligned} \tag{S9}$$

Solving for the extrema and simplifying, we find that the dynamic ratios of B and of P are given by:

$$
\begin{aligned}
\frac{B^{\max}}{B^{\min}} =& \frac{C - P_0^{\text{fp}}}{bB_0^{\text{fp}}} \exp\left[\frac{C - P_0^{\text{fp}}}{bB_0^{\text{fp}}}\right], \\
\frac{P^{\max}}{P^{\min}} =& \frac{C - bB_0^{\text{fp}}}{P_0^{\text{fp}}} \exp\left[\frac{C - bB_0^{\text{fp}}}{P_0^{\text{fp}}}\right].
\end{aligned} \tag{S10}
$$

To understand the growth of the dynamic ratio of B for large α/δ and of P for small α/δ, we first recognize that the positivity of the dynamic ratios implies $C > P_0^{\text{fp}}$ and $C > bB_0^{\text{fp}}$, so that the dynamic ratios can be approximated as

$$
\begin{aligned}
\frac{B^{\max}}{B^{\min}} \approx& \frac{C}{bB_0^{\text{fp}}} \exp\left[\frac{C}{bB_0^{\text{fp}}}\right], \\
\frac{P^{\max}}{P^{\min}} \approx& \frac{C}{P_0^{\text{fp}}} \exp\left[\frac{C}{P_0^{\text{fp}}}\right].
\end{aligned} \tag{S11}
$$

Substituting in from Eqs. (S9), we find

$$
\begin{aligned}
\frac{B^{\max}}{B^{\min}} \approx& \frac{bB^{\max} + P_0^{\text{fp}}}{bB_0^{\text{fp}}} \exp\left[\frac{bB^{\max} + P_0^{\text{fp}}}{bB_0^{\text{fp}}}\right], \\
\frac{P^{\max}}{P^{\min}} \approx& \frac{P^{\max} + bB_0^{\text{fp}}}{P_0^{\text{fp}}} \exp\left[\frac{P^{\max} + bB_0^{\text{fp}}}{P_0^{\text{fp}}}\right].
\end{aligned} \tag{S12}
$$

Finally, substituting in for the dynamical fixed point values (Eqs. (S1)), we arrive at

$$
\begin{aligned}
\frac{B^{\max}}{B^{\min}} \approx& \left(\frac{B^{\max}}{B_0^{\text{fp}}} + \frac{\alpha - \mu}{\delta}\right) \exp\left[\left(\frac{B^{\max}}{B_0^{\text{fp}}} + \frac{\alpha - \mu}{\delta}\right)\right], \\
\frac{P^{\max}}{P^{\min}} \approx& \left(\frac{P^{\max}}{P_0^{\text{fp}}} + \frac{\delta}{\alpha - \mu}\right) \exp\left[\left(\frac{P^{\max}}{P_0^{\text{fp}}} + \frac{\delta}{\alpha - \mu}\right)\right].
\end{aligned} \tag{S13}
$$

For large α (i.e. $\alpha \gg \delta$), the dynamic ratio of B is therefore dominated by $e^{\alpha/\delta}$. Similarly, for small α (i.e. $\alpha - \mu \ll \delta$), the dynamic ratio of P is dominated by $e^{\delta/(\alpha-\mu)}$.

To find the crossover between the two dynamic regimes (one where the dynamic ratio of B is large, and the other where the dynamic ratio of P is large), we solve for

$$
\frac{B^{\max}/B^{\min}}{P^{\max}/P^{\min}} = 1 \qquad \text{(at crossover).} \tag{S14}
$$

Starting from Eq. (S11), this can be approximated as

$$
\frac{P_0^{\text{fp}}}{bB_0^{\text{fp}}} \approx \exp\left[C\left(\frac{1}{bB_0^{\text{fp}}} - \frac{1}{P_0^{\text{fp}}}\right)\right] \qquad \text{(at crossover).} \tag{S15}
$$

While the precise value of C depends on the particular initial conditions chosen, it can be estimated by its value at the fixed point, $C^{\text{fp}} = P_0^{\text{fp}} + bB_0^{\text{fp}}$. This yields

$$
\frac{P_0^{\text{fp}}}{bB_0^{\text{fp}}} \approx \exp\left[\frac{P_0^{\text{fp}}}{bB_0^{\text{fp}}} - \frac{bB_0^{\text{fp}}}{P_0^{\text{fp}}}\right] \qquad \text{(at crossover).} \tag{S16}
$$

The equation $x = \exp\left[x - x^{-1}\right]$ is solved by $x = 1$. Therefore, the crossover occurs at

$$P_0^{\mathrm{fp}} \approx b B_0^{\mathrm{fp}} \qquad \text{(at crossover)}, \tag{S17}$$

or, substituting in for the dynamical fixed point values (Eqs. (S1)),

$$\alpha - \mu \approx \delta \qquad \text{(at crossover)}. \tag{S18}$$

Supplemental References

[S1] Sarah Camara-Wilpert, David Mayo-Muñoz, Jakob Russel, Robert D. Fagerlund, Jonas S. Madsen, Peter C. Fineran, Søren J. Sørensen, and Rafael Pinilla-Redondo. Bacteriophages suppress CRISPR–Cas immunity using RNA-based anti-CRISPRs. Nature, 623(7987):601–607, 2023.

[S2] Ofer Kimchi, Yigal Meir, and Ned S Wingreen. Lytic and temperate phage naturally coexist in a dynamic population model. The ISME Journal, 18(May):1–5, 2024.
